# The diagnosis and management of ADHD (Attention Deficit Hyperactivity Disorder) in children and young people: a commentary on current practice and future recommendations

**DOI:** 10.3399/bjgpopen20X101043

**Published:** 2020-03-04

**Authors:** Zara Bowling, Alexandra Nettleton

**Affiliations:** 1 Core Psychiatry Trainee, Psychiatry Department, Peninsula Postgraduate Medical Education, Plymouth, UK; 2 Paediatrics Registrar, Paediatrics Department, Severn NHS Trust, Bristol, UK

**Keywords:** Attention deficit disorder with hyperactivity, ADHD, Child health, Mental health, general practice, primary health care

Attention deficit hyperactivity disorder (ADHD) is a behavioural disorder with symptoms of hyperactivity, impulsivity, and inattention.^1^ It is estimated that the prevalence of ADHD worldwide is 5.29% in children and adolescents, and it is more common in males.^2^ ADHD is known as Hyperkinetic Disorder by the World Health Organization (WHO), and over a person’s lifetime, the symptoms and impact of the condition may vary considerably.^2^

Guidelines state symptoms must meet either International Statistical Classification of Diseases and Related Health Problems (ICD-10) or Diagnostic and Statistical Manual of Mental Disorders, 5th Edition (DSM-5) criteria; cause moderate to severe functional impairment; and occur in more than one setting.^1–4^ Assessment for ADHD includes a thorough clinical examination, and a full developmental and psychosocial history, alongside information gathering from the family and school.^1^ ADHD must be diagnosed by a paediatrician, psychiatrist, or ADHD specialist.

Early diagnosis and intervention improves educational outcomes for children and is important for their social development.^1^ The chronic nature of ADHD means that symptoms often persist through to adulthood, initially affecting a child or adolescent’s schooling, friendships, and daily life, and later on disrupting work and relationships. It is associated with significant impairment worldwide.^2^

The Care Quality Commission has identified long waiting times between referral and diagnosis, with waits for treatment of up to 18 months.^5^ A report by Young Minds in 2018 found the majority of children and young people were waiting 5 months to a year for an initial assessment, and over 50% of children requiring treatment then had to wait at least 6 months before treatment was initiated.^6^ Evidence indicates treatment begins within 4 weeks of being seen in Child and Adolescent Mental Health Services (CAMHS) for about 14% of children and young people at present.^6^ Internationally, experts worldwide have released a consensus statement sharing their concerns: that despite effective treatment being available for children and young people, the majority of these individuals do not receive adequate and timely treatment.^7^

National Institute for Health and Care Excellence (NICE) guidance gives clear recommendations for initial diagnosis, ongoing symptom management, and transition of care for children and young people with ADHD.^1^ A joint service, multicentre research project in the South West between CAMHS and Paediatrics compared current practice to NICE recommendations. The results showed that the majority of children waited more than 6 months to be seen by CAMHS following primary care referral. The 2018 green paper, *Transforming Children and Young People’s Mental Health Provision,* proposes a maximum waiting time of 4 weeks from referral to CAMHS.^6^ Most families received no written information about ADHD at diagnosis, whereas NICE guidance states that support should be offered to families, and parents should be asked how the diagnosis has affected them and how they can be supported.^1^

A number of international recommendations to primary care and CAMHS arose from the multicentre study that would improve service provision to children and young people with ADHD. The overall recommendation is a requirement for improved adherence to NICE guidance in order to promote equality across healthcare services for children and young people.

Specifically, the international recommendations are:

1. to increase the screening tool availability in primary care and CAMHS, in order to improve the diagnostic process. Diagnosis in primary care with access to secondary care specialist advice would reduce workload and speed up treatment initiation. NICE guidance recommends the use of the Connor’s screening questionnaire, which has proven to be useful as an adjunct in the diagnosis of ADHD.^1^
2. that information and education needs to be provided to families both within primary care and CAMHS. A package of care could be initiated on diagnosis of ADHD, to include written information, and referral to the specialist nurse and specialist parenting groups. NICE guidance suggests psychoeducation, specialist parenting, and support for the child or young person following diagnosis.^1^
3. that effective communication between primary care and CAMHS could enable earlier diagnosis and improve the continuity of care between services. GPs are often responsible for the ongoing prescription of ADHD medication, physical monitoring, and follow-up once ADHD is diagnosed by secondary care services. NICE emphasises the importance of continuity of care for children and young people.^1^

Increased demands in combination with limited capacity and resources in primary care and CAMHS make reducing waiting times between referral and initiation of treatment a challenge.^8^ At present, because over 80% of children and young people referred to secondary care with suspected ADHD do receive a diagnosis, there are high numbers of individuals currently untreated.^2^ Improved screening for ADHD in primary care would reduce demand on secondary services and enable treatment to be initiated sooner for children and young people.
